# A Case Study of Deep Vein Thrombosis of the Right Internal Jugular Vein in a Healthy 21-Year-Old Male

**DOI:** 10.1155/2016/7654749

**Published:** 2016-09-20

**Authors:** Javier Corral, Geri Villanueva

**Affiliations:** ^1^Division of Hematology/Oncology, Department of Internal Medicine, Paul L. Foster School of Medicine, Texas Tech University Health Care Center, 4800 Alberta Avenue, El Paso, TX 79905, USA; ^2^Paul L. Foster School of Medicine, Texas Tech University Health Care Center, 5001 El Paso Drive, El Paso, TX 79905, USA

## Abstract

We are reporting a case of a healthy 21-year-old male, with no significant past medical history, who was found to have an incidental nonocclusive deep vein thrombosis in the right internal jugular vein detected on a head MRI previously ordered for work-up of headaches. A follow-up upper extremity venous Doppler ultrasound confirmed the presence of a partially occlusive deep vein thrombosis in the right jugular vein. The case presented is unique for the reason that the patient is young and has no prior risk factor, personal or familial, for venous thrombosis except for associated polycythemia on clinical presentation.

## 1. Introduction 

Isolated internal jugular vein (IJV) thrombosis is a rare condition that is underdiagnosed and associated with significant morbidity such as pulmonary embolism and postthrombotic syndrome. The most common causes of IJV thrombosis are cancer, central venous catheter, and ovarian hyperstimulation syndrome. Patients may present with a painful swollen neck or may be asymptomatic.

There are no set guidelines for the treatment for IJV thrombosis but most reported cases of IJV thrombosis are treated similarly to lower extremity DVTs, primarily with a 3–6-month course of anticoagulation therapy, and in rare selected cases with initial thrombolysis, venous thrombectomy, or placement of a superior vena cava filter when anticoagulation is contraindicated.

## 2. Case Report

The patient is a 21-year-old male who was referred by his primary care physician (PCP) to the Emergency Department (ED) for an abnormal head MRI, done as outpatient, which revealed partial occlusion of the right IJV. A couple of months priorly, the patient saw his PCP complaining of headaches which prompted his PCP to order an MRI of the brain. Before the MRI was performed, the patient acquired new corrective eye glasses which caused his headaches to resolve. However, the patient still decided to go ahead with his scheduled head MRI.

The patient on presentation at the ED denied any headaches, chest pain, palpitations, shortness of breath, right neck pain or swelling, or right arm pain or swelling.

The patient reports that he is in good health and exercises regularly. He has no previous injuries and denies tobacco, alcohol, and drug use. He regularly lifts heavy weights and uses a nutritional supplement containing calcium fructopyranose borate, which is a patented form of boron. There is no family history of hypercoagulable states, clotting disorders, or deep vein thrombosis (DVT).

Examination revealed an otherwise fit-looking young man without any significant physical findings. MRI of the brain did not show any intracranial pathology but incidentally revealed partial occlusion of the right internal jugular vein. An upper extremity venous Doppler ultrasound study was then performed which confirmed the presence of a partially occlusive deep vein thrombosis in the right IJV. The age of the thrombus was not known.

Investigations, including complete blood count, coagulation studies, and renal function tests, yielded normal results with the exception of elevated red blood cell count, hemoglobin and hematocrit which were 6.33 million cells per microliter, 18.1 g/dL and 52.3 percent, respectively. In the ED, the patient was then placed on Xarelto (rivaroxaban) 20 mg orally daily and referred to the hematology clinic for further evaluation. Work-up for thrombophilic disorders included homocysteine, protein C, antithrombin III, and anti-cardiolipin antibody levels which were all within the normal range. Protein S was slightly elevated by about two percent above normal. Lupus anticoagulant, factor V Leiden mutation, JAK2 mutation, and G20210A mutation in the prothrombin/factor II gene were not detected. Erythropoietin levels were also within the normal range.

After three months of taking Xarelto, the patient underwent a repeat right upper extremity venous ultrasound study and results were negative for DVT and Xarelto was discontinued. Patient also at the time of his initial hematological evaluation was counseled to stop his boron containing nutritional supplements and repeat hemoglobin and hematocrit levels two months later revealed that his hemoglobin and hematocrit had decreased to 16.6 and 49.8, respectively.

## 3. Discussion

Isolated IJV thrombosis is a rare entity and can occur in association with upper extremity deep vein thrombosis (UEDVT), a thrombosis of the brachiocephalic, subclavian, axillary, brachial, ulnar, or radial veins, and is associated with a significant risk for pulmonary embolism and postthrombotic syndrome.

In one retrospective analysis of 1,948 patients with DVT, isolated IJV thrombosis was diagnosed in 29 patients (1.5%). IJV thrombosis was most commonly associated with upper extremity thrombosis in 72% of the cases compared to 28% of isolated cases. It was more common in women (76%) and the median age was 46 years [1]. In another retrospective study of 210 patients found to have UEDVT or IJV thrombosis, 21 patients had IJV thrombosis only and 61 patients had concomitant IJV thrombosis and UEDVT. The condition was also more common in women and the mean age was 67 ± 18 [2].

In both studies, the primary treatment was initial systemic anticoagulation therapy with either unfractionated or low molecular weight heparin followed by oral anticoagulation with vitamin K antagonist and in some cases no treatment was given. In few selected cases where anticoagulation was contraindicated, a superior vena cava filter was placed.

Our patient, a young and active male who regularly lifts weights, developed a thrombus in the IJV only and not in the upper extremity veins to suggest this was a case of Paget-Schroetter syndrome. Patients with effort thrombosis or Paget-Schroetter syndrome are usually young and have no major illnesses or risk factors for cardiovascular diseases [3, 4]. These patients often develop spontaneous upper extremity deep vein thrombosis (UEDVT) in their dominant arm after strenuous activity, which can cause microtrauma to the intimal layer of the vein leading to activation of coagulation cascade. Paget-Schroetter syndrome is also related to thoracic outlet obstruction due to anatomical compression of veins or costoclavicular crowding from bone anomalies or hypertrophied muscles amongst athletes who do heavy weight lifting or completely abduct their arms.

Our patient's only other clinical manifestation on presentation was polycythemia. He was taking a supplement that contains boron, a chemical element found in many plant-based foods which have also been associated with increased androgen levels [5, 6]. It is well established that androgen therapy can lead to increase in erythropoiesis. There are also reports of androgen abuse linked to strokes and venous thrombosis in young healthy athlete [7, 8]. Although his testosterone levels were not measured at the time of his ED visit, or initial hematological evaluation, they were measured 6 weeks after the patient was counseled to stop the boron containing supplement and his free testosterone levels were then measured and were found to be above the normal levels at 159.5 pg/mL (range 35.0–155.0 pg/mL). Furthermore, his hemoglobin and hematocrit levels decreased in 6 weeks after discontinuation of the boron containing supplement.

Although the patient did not receive initial systemic anticoagulation therapy with either unfractionated or low molecular weight heparin, he responded favorably to upfront oral anticoagulation therapy with Xarelto, a factor Xa inhibitor, with complete resolution of his thrombosis.

## 4. Conclusion

This case is an unusual presentation of an isolated IJV thrombosis in an otherwise young healthy man with no risk factors other than taking boron containing supplement and associated polycythemia which responded favorably to primary therapy with an oral factor Xa inhibitor. We suspected that the cause of his IJV thrombosis was either an indirect cause of the intake of boron containing supplement and its associated polycythemia, or an idiopathic thrombosis, rather than a result of Paget-Schroetter syndrome, or another underlying primary or secondary hypercoagulable condition. To this date, the patient has had no relapse and his hematocrit and hemoglobin levels have remained near the normal range. A possible link between boron containing nutritional supplements, polycythemia, and thrombogenesis may warrant further clinical investigations.

## References

[1] Gbaguidi X., Janvresse A., Benichou J., Cailleux N., Levesque H., Marie I. (2011). Internal Jugular vein Thrombosis: outcome and risk factors. *Quarterly Journal of Medicine*.

[2] Ascher E., Salles-Cunha S., Hingorani A. (2005). Morbidity and mortality associated with internal jugular vein thromboses. *Vascular and Endovascular Surgery*.

[3] Hughes E. S. R. (1948). Venous obstruction in the upper extremity. *British Journal of Surgery*.

[4] Illig K. A., Doyle A. J. (2010). A comprehensive review of Paget-Schroetter syndrome. *Journal of Vascular Surgery*.

[5] Naghii M. R., Mofid M., Asgari A. R., Hedayati M., Daneshpour M.-S. (2011). Comparative effects of daily and weekly boron supplementation on plasma steroid hormones and proinflammatory cytokines. *Journal of Trace Elements in Medicine and Biology*.

[6] Naghii M. R., Samman S. (1997). The effect of boron supplementation on its urinary excretion and selected cardiovascular risk factors in healthy male subjects. *Biological Trace Element Research*.

[7] Frankle M. A., Eichberg R., Zachariah S. B. (1988). Anabolic androgenic steroids and a stroke in a athlete: case report. *Archives of Physical Medicine and Rehabilitation*.

[8] Jaillard A. S., Hommel M., Mallaret M. (1994). Venous sinus thrombosis associated with androgens in a healthy young man. *Stroke*.

